# Transcriptional Response of Rice Phytocystatin Family Genes to Cold Stress

**DOI:** 10.3390/genes17091037

**Published:** 2026-08-29

**Authors:** Mingbo Li, Tingting Yang, Deyu Kong, Jin Xu

**Affiliations:** 1 College of Agronomy and Biotechnology, Yunnan Agricultural University, Kunming 650201, China; ynlimingbo@163.com (M.L.); tytingtingy@163.com (T.Y.); 2023210179@stu.ynau.edu.cn (D.K.); 2 Rice Research Institute, Yunnan Agricultural University, Kunming 650201, China

**Keywords:** cold stress, gene expression, phytocystatins, rice

## Abstract

**Background/Objectives:** Phytocystatins are plant-specific inhibitors of papain-like cysteine proteinases that participate in plant development and responses to biotic and abiotic stresses. The roles of rice (*Oryza sativa* L.) phytocystatins under cold stress remain poorly understood. Xiaomagu (XMG), a cold-tolerant japonica rice landrace, provides valuable material for exploring phytocystatin transcript responses to low-temperature stress. This study characterizes 12 rice phytocystatin genes isolated from XMG and investigates their transcript profiles under cold and abscisic acid (ABA) treatments. **Methods:** Twelve phytocystatin genes were isolated from the cold-tolerant japonica rice landrace Xiaomagu. Sequence characterization and conserved domain analysis were performed for these phytocystatin family members. Real-time quantitative PCR was performed to examine transcript abundance of phytocystatin genes: low-temperature stress was applied to leaf and root tissues, while ABA treatment was carried out for leaf tissues only. **Results:** Conserved domain analysis revealed structural differences among the identified phytocystatin members. Real-time quantitative PCR detected distinct transcriptional responses to cold stress among these phytocystatin genes. In leaves, *OsCST1*, *OsCST8*, and *OsCST12* were up-regulated, whereas *OsCST11* was down-regulated. In roots, *OsCST2*, *OsCST3*, *OsCST6*, and *OsCST12* showed increased transcript levels, while *OsCST1* and *OsCST11* were down-regulated. *OsCST2*, *OsCST6*, and *OsCST11* exhibited significantly altered transcript abundance under ABA treatment. **Conclusions:** This study characterizes transcript-level responses of 12 phytocystatin genes from the cold-tolerant rice landrace Xiaomagu (XMG) under cold and ABA treatments. The detected expression changes reflect stress-responsive transcriptional regulation of rice phytocystatin family genes. Further in planta functional assays and comparisons with cold-sensitive genotypes are needed to elucidate their precise roles in rice cold adaptation.

## 1. Introduction

Rice is native to tropical and subtropical regions and is inherently sensitive to low-temperature conditions. Cold stress frequently disrupts normal rice growth and developmental processes, severely restricting crop productivity in temperate and high-altitude planting regions. To cope with low-temperature stress, plants dynamically reprogram diverse primary metabolic pathways to facilitate cold adaptation. The ubiquitin-proteasome system constitutes a major route for controlled protein turnover during plant abiotic stress adaptation [1]. As specific inhibitors targeting papain-like cysteine proteinases, phytocystatin family genes play potential regulatory roles in cysteine-protease-mediated protein metabolism. However, their cold-responsive characteristics and biological functions in rice under low-temperature stress remain poorly documented and largely unclarified.

Phytocystatins are plant-specific cysteine proteinase inhibitors (CPIs), which contain the plant-specific conserved LARFAV motif in peptide sequences. The inhibitory activity of phytocystatins relies on three structurally conserved motifs with distinct functional roles. The central conserved QxVxG motif (with a core Q-V-G sequence) functions as the primary functional domain that forms a hydrophobic wedge structure, which sterically inserts into the active site of papain-like cysteine proteases and reversibly inhibits protease hydrolytic activity [2,3]. The conserved C-terminal tryptophan residue within the PW hairpin loop contributes to the structural stabilization of the inhibitor–enzyme complex by hydrophobic anchoring to the active-site pocket of target proteases, which is essential for complete inhibitory competence [3]. However, phytocystatins share identical inhibitory mechanisms with animal homologs, owing to their similar tertiary structures containing the conserved QxVxG inhibitory motif and a conserved tryptophan residue at the peptide C-terminus [4].

Research data on phytocystatins imply their crucial roles as defense and proteolysis regulators in plants by inhibiting papain-like cysteine proteinase activity [5]. Phytocystatins participate in diverse biological processes, including seed germination [5,6,7], seedling growth and development [8], and programmed cell death [9,10]. Transgenic tobacco plants constitutively overexpressing the rice cystatin OC-I displayed pleiotropic phenotypes such as accelerated growth and increased flower and seed production [11]. Beyond developmental roles, phytocystatins contribute substantially to plant biotic defense against herbivores and pathogens. For example, the wheat phytocystatin TaMDC1 inhibits mycelial growth of the snow mold *Microdochium nivale* in vitro, and its ectopic expression in tomato confers enhanced resistance against fungi, bacteria, and insects [12].

Besides biotic-stress responses, accumulating evidence indicates that phytocystatins also participate in plant adaptation to multiple abiotic stresses, including high salinity, drought, and low-temperature stress. For instance, the apple cystatin gene *MpCYS5* from *Malus prunifolia* is induced by high-salinity stress and modulates reactive oxygen species accumulation under stress conditions [13]. Low-temperature-responsive gene expression constitutes a core component of plant cold-adaptation mechanisms. Cold exposure remodels plant physiological status and triggers transcriptional reprogramming of phytocystatin family genes to improve stress tolerance. Several studies have reported stress-dependent transcriptional alterations of phytocystatin genes under cold conditions. The barley cysteine proteinase-inhibitor gene *Icy*, expressed across multiple organs, shows elevated transcript abundance in vegetative tissues upon cold shock, and its recombinant protein effectively inhibits papain and ficin activities [14]. In wheat, both mRNA and protein abundances of *TaMDC1* increase during cold acclimation [15]. Two maize cystatin genes are also cold-stress-inducible at the transcript level [16]. Beyond transcript-level responses, transgenic assays have provided direct functional evidence for phytocystatins in stress tolerance. Transgenic *Arabidopsis thaliana* plants overexpressing *AtCYSa* or *AtCYSb* acquire improved tolerance to multiple adverse conditions including cold stress; additionally, *AtCYSa* transcript levels are responsive to exogenous ABA treatment [17].

Twelve phytocystatin genes were identified by genome-wide screening of the japonica reference cultivar Nipponbare; By comparison, orthologous cystatin genes from indica rice harbor minor amino-acid polymorphisms that do not map to the conserved functional motifs [18]. Previous studies have profiled rice cystatin gene expression across diverse tissues and developmental stages, as well as transcript-level changes under adverse conditions such as high salinity and wounding [19]. Nevertheless, cold-stress-dependent expression responses of rice cystatin family members remain poorly documented. To date, functional assessment of rice cystatins under cold conditions is limited. Heterologous overexpression of rice *OC-I* in transgenic tobacco alleviated photosynthetic impairment under chilling stress relative to wild-type controls [20]; however, this protective effect was observed in a heterologous system and has not been validated in rice plants.

In the present study, we analyzed sequence variation in phytocystatin genes within Xiaomagu (XMG), a japonica rice landrace adapted to alpine rice-growing zones above 2300 m in Yunnan Province, China. This landrace exhibits prominent cold-tolerance performance under low-temperature conditions [21]. We further characterized transcript-level expression patterns of phytocystatin family genes under cold stress, aiming to screen candidate genes for future functional investigations into rice cold-adaptation mechanisms.

## 2. Materials and Methods

### 2.1. Plant Material Collection

The cold-tolerant rice landrace Xiaomagu (XMG) was used as experimental material. Rice seedlings were cultivated in programmable plant growth chambers (Shanghai Yiheng Scientific Instrument Co., Ltd., Shanghai, China) under a 12 h light/12 h dark photoperiod, 70 ± 5% relative humidity, and a constant light intensity of 250 μmol m^−2^ s^−1^. For each biological replicate, 40 seeds were sown. At the four-leaf stage, uneven and phenotypically inconsistent seedlings were removed, and 27 uniformly growing individuals per replicate were retained for subsequent cold and ABA stress treatments.

For cold stress treatment, four-leaf-stage seedlings were exposed to a constant low temperature of 8 °C. Leaf and root tissues were separately collected at 0, 4, 12, 24, and 48 h post-treatment. To reduce systematic errors, two independent incubators were used alternately for all replicates, and seedling trays were re-randomized after each sampling to minimize microenvironmental positional differences.

For ABA treatment, ABA powder (Solarbio Science & Technology Co., Ltd., Beijing, China) was dissolved in absolute ethanol to prepare a stock solution and diluted with deionized water to a final concentration of 75 μmol L^−1^. Uniform four-leaf-stage seedlings were evenly sprayed with the ABA working solution. Seedlings sprayed with an equal volume of absolute ethanol-containing deionized water with matched solvent concentration were set as the solvent control to eliminate ethanol effects on seedling growth.

All treatments and controls were performed with three independent biological replicates. Seedlings with consistent growth were selected, and leaf and root tissues at identical positions were separately sampled and pooled into individual mixed samples. All sampling was conducted at a fixed time during the light phase. All samples were immediately snap-frozen in liquid nitrogen and stored at −80 °C for subsequent molecular experiments.

### 2.2. RNA Extraction and cDNA Synthesis

Total RNA was isolated from rice leaf and root tissues using the RNA-prep Pure Plant Kit (Tiangen Biotech Co., Ltd., Beijing, China) Agarose gel electrophoresis was performed to detect RNA integrity, which was used to evaluate the overall purity and quality of the extracted total RNA. First-strand cDNA (Tiangen Biotech Co., Ltd., Beijing, China) was synthesized strictly in accordance with the manufacturer’s standard protocols. No-reverse-transcription (NRT) controls and no-template controls (NTC) were included in each round of cDNA synthesis and qPCR amplification to exclude potential genomic DNA contamination and reagent contamination.

### 2.3. Gene Cloning

Specific primer pairs for 12 genes of the rice phytocystatin gene family were designed based on sequences retrieved from the NCBI database (Table S1). High-fidelity PCR was performed using cDNA templates isolated from the cold-tolerant rice landrace Xiaomagu (XMG) to amplify the full-length coding sequences of these 12 genes. PCR amplification was carried out in a 20 µL reaction system consisting of 10 µL 2× Taq PCR Master Mix (BioNTech (Shanghai) Co., Ltd., Shanghai, China), 2 µL forward primer (2 µM), 2 µL reverse primer (2 µM) (synthesized by BioN Tech (Shanghai) Co., Ltd.), 1 µL cDNA template, and 5 µL nuclease-free water. The PCR program was set as follows: initial denaturation at 94 °C for 5 min; 30 cycles of 94 °C for 30 s, 66 °C for 45 s, and 72 °C for 45 s; followed by a final extension at 72 °C for 5 min. Amplified PCR products were purified using the StarPrep Gel Extraction Kit (GenStar Biosolutions Co., Ltd., Beijing, China) and ligated into the pMD18-T cloning vector (Takara Bio Inc., Kusatsu, Shiga, Japan) according to the manufacturer’s instructions. Recombinant vectors were transformed into Escherichia coli DH5α competent cells (Takara Bio Inc., Kusatsu, Shiga, Japan), and positive clones were screened for Sanger sequencing verification. Sequencing results were aligned with the rice reference genome sequences to systematically analyze the sequence characteristics of the rice phytocystatin gene family.

### 2.4. Real-Time Quantitative PCR

Real-time quantitative PCR (qRT-PCR) was performed on a MyGo Pro PCR system (IT-IS International Ltd., Stokesley, UK) to detect the transcript levels of phytocystatin family genes under cold and ABA treatments. TSINGKE 2 × TSINGKE Master qPCR Mix (TSINGKE Biological Technology Co., Ltd., Beijing, China) (contains SYBR Green I but no intrinsic ROX reference dye) was used for real-time quantitative PCR (qPCR) assays. All qPCR primer sequences are listed in Table S2. The reactions were carried out in a final volume of 10 μL, containing 5 μL of TSING-KE 2× qPCR Mix (SYBR Green I), 0.4 μL of each reverse and forward primer (10 μM), 0.2 μL of 50× ROX, 1 μL of cDNA, and 3 μL of ddH_2_O. The amplification procedure was set as follows: 95 °C for 1 min, followed by 40 cycles of 95 °C for 10 s, 60 °C for 20 s, and 72 °C for 20 s.

The expression stability of candidate reference genes under cold-stress treatments in leaf and root tissues was separately assessed using the BestKeeper algorithm, and the calculated SD values are provided in the Supplementary Materials. In leaf samples, both *Actin* (SD = 0.415) and *FDH* (SD = 0.659) displayed SD values below 1, showing high expression stability suitable for reference gene selection, Accordingly, *FDH* and *Actin* were chosen as dual reference genes for leaf samples. For root samples, *FDH* had an SD value of 0.677, whereas *Actin* showed a higher SD of 0.722, indicating greater expression variation in *Actin* in roots under cold-stress conditions. Thus, only *FDH* was used as the reference gene for root-sample normalization. All leaf and root samples were analyzed in three biological replicates, and each biological replicate included three technical replicates. Relative gene expression levels were calculated using the $2^{-\Delta \Delta C_{t}}$ method. All primer pairs used for qRT-PCR amplification in this study were referenced from a previously published study [19].

### 2.5. RT-PCR Analysis

Semi-quantitative RT-PCR was adopted for qualitative detection of low-abundance target gene transcripts. The 20 μL RT-PCR reaction system contained 10 μL 2× Taq PCR StarMix (GenStar Biosolutions Co., Ltd., Beijing, China), 0.6 μL of each forward and reverse primer (10 μM), 1 μL cDNA template, and 7.8 μL ddH_2_O. The thermal cycling program was set as follows: initial denaturation at 94 °C for 2 min, followed by 25–40 cycles of 94 °C for 30 s, 60 °C for 30 s, and 72 °C for 30 s, with a final extension step at 72 °C for 5 min. *OsFDH* was used as the internal reference gene for semi-quantitative detection. Subsequently, PC R products were separated by 2% agarose gel electrophoresis at 120 V for 20 min, and amplified bands were visualized and analyzed using a gel imaging system (Shanghai Tanon Science & Technology Co., Ltd., Shanghai, China).

### 2.6. Sequence Alignment and Phylogenetic Tree Construction

The amino acid sequences of rice phytocystatins in XMG were aligned using the MUSCLE algorithm in MEGA 11, and a phylogenetic tree was constructed by the neighbor-joining (NJ) method. Bootstrap analysis was calculated for 1000 replicates [22]. Locus IDs of identified rice phytocystatin genes are summarized in Table S3. Gene sequences and annotation information were verified against NCBI Gene database, Rice Genome Annotation Project (RGAP 7.0), and relevant published literature.

### 2.7. Tissue-Specific Expression Profiling of Gene Family

Gene expression data acquisition and heatmap analysis: for tissue expression profiling of phytocystatin family genes, publicly available transcriptome data of rice cultivar Nipponbare were retrieved from the Rice Expression Database (RED) [23]. The dataset covers multiple tissues, including roots, stems, leaves, panicles, anthers, pistils, and seeds, across diverse developmental stages. The raw data downloaded from RED are provided in Supplementary File S1. Heatmaps visualizing the expression patterns of the 12 identified phytocystatin genes were generated using Heml software 1.0 with default parameters [24].

### 2.8. Statistical Analysis

Raw data sorting, statistical analysis and graphical visualization in this study were completed using Excel 2019, DPS 18.10, and GraphPad Prism 9.5. Excel was used for raw data collation and basic numerical calculation; DPS 18.10 was employed for statistical analyses including one-way ANOVA, Duncan’s multiple range test and correlation analysis; GraphPad Prism 9.5 was used for all experimental data plotting. A significance threshold of *p* < 0.05 was adopted for all statistical tests to guarantee credible analytical results.

## 3. Results

### 3.1. Coding Sequence Variation in Phytocystatins Family Genes in the Genome of an Highly Cold-Tolerant Rice Variety

A total of 12 rice phytocystatin genes were isolated from Xiaomagu (XMG), a highly cold-tolerant japonica rice landrace originating from high-altitude rice-growing regions in Yunnan, China. Comparative sequence analysis with the 12 predicted phytocystatin homologs from the reference japonica rice cultivar Nipponbare identified four distinct allelic variants in the XMG genome. *OsCST1* carries a synonymous nucleotide substitution at the 93 bp position. Amino-acid variations were detected in *OsCST3* and *OsCST11*. Specifically, *OsCST3* harbors a 4-amino-acid deletion spanning residues 151–154 aa (VVFP), while *OsCST11* contains a single non-synonymous substitution at amino-acid position 27, leading to a valine-to-leucine (V → L) alteration. These variable amino-acid sites do not overlap with the conserved functional domains of phytocystatin proteins.

**Figure 1:**
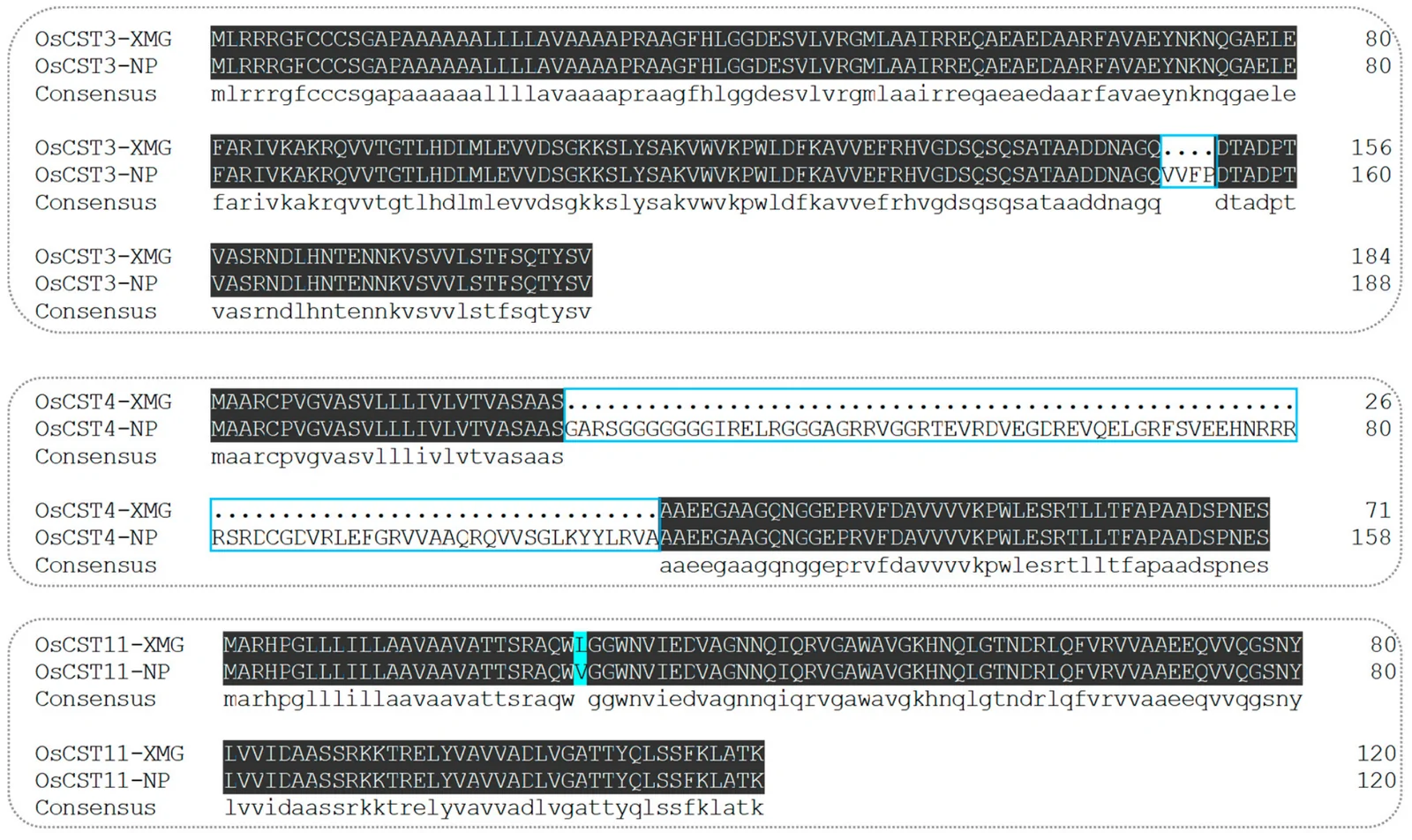
Alignment of the phytocystatins from different rice varieties. The deduced protein sequence of *OsCST3*, *OsCST4* and *OsCST11* in rice (cv. XMG) was compared with their counterparts in Nipponbare, respectively.

Notably, the *OsCST4* coding sequence cloned from XMG is only 216 bp, encoding a truncated protein of 71 amino acids, with an 87-amino-acid sequence loss compared with its Nipponbare homolog. This unique structural variation indicates obvious genetic divergence of *OsCST4* between the cold-tolerant landrace XMG and the reference cultivar Nipponbare, which may contribute to its distinct transcriptional and potential functional characteristics (Figure 1).

**Figure 2:**
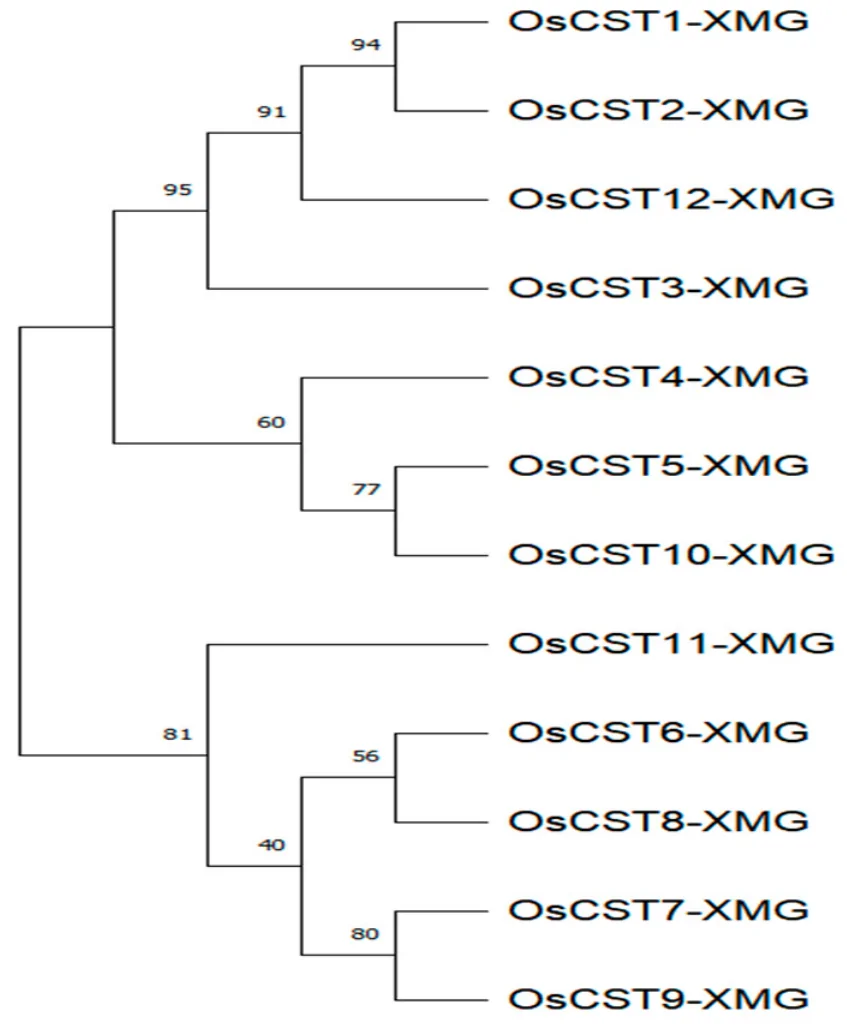
Phylogenetic tree of phytocystatin proteins from rice landrace Xiaomagu (XMG). Amino-acid sequences were aligned using the MUSCLE algorithm implemented in MEGA 11. Ambiguous alignment sites and gaps were trimmed, and a neighbor-joining (NJ) phylogenetic tree was generated with 1000 bootstrap replicates. Numbers at nodes represent bootstrap support values.

Due to sequence variations among phytocystatin genes in the XMG genome, an unrooted phylogenetic tree was constructed using the 12 phytocystatin protein sequences from XMG. The phylogenetic tree divided the rice phytocystatin family into three subfamilies. The first subfamily contained four members, including *OsCST1*, *OsCST2*, *OsCST3*, and *OsCST12*; the second subfamily comprised *OsCST4*, *OsCST5*, and *OsCST10*; the third subfamily included *OsCST6*, *OsCST7*, *OsCST8*, *OsCST9*, and *OsCST11* (Figure 2).

### 3.2. Transcriptional Variation in the Rice Phytocystatins Family Genes Under Low Temperature Stress

To obtain a detailed overview of transcriptional changes in all rice cystatin genes under cold-stress conditions, we quantified the mRNA levels of the 12 rice phytocystatin genes in leaves and roots at multiple cold-stress time points using real-time quantitative PCR. No amplification products were detected for five genes: *OsCST4*, *OsCST5*, *OsCST7*, *OsCST9*, and *OsCST10*. We therefore further assessed transcript presence for these five genes using conventional semi-quantitative RT-PCR combined with agarose gel electrophoresis, employing leaf samples from cold and ABA treatments. Target amplicons for *OsCST5*, *OsCST9*, and *OsCST10* were only detectable at >35 amplification cycles, indicative of extremely low transcript abundance. Results derived from these three genes should therefore be interpreted with caution, as their very low expression levels limit robust biological inference. While the expression of *OsCST4* and *OsCST7* remained un-detectable throughout the treatment.

**Figure 3:**
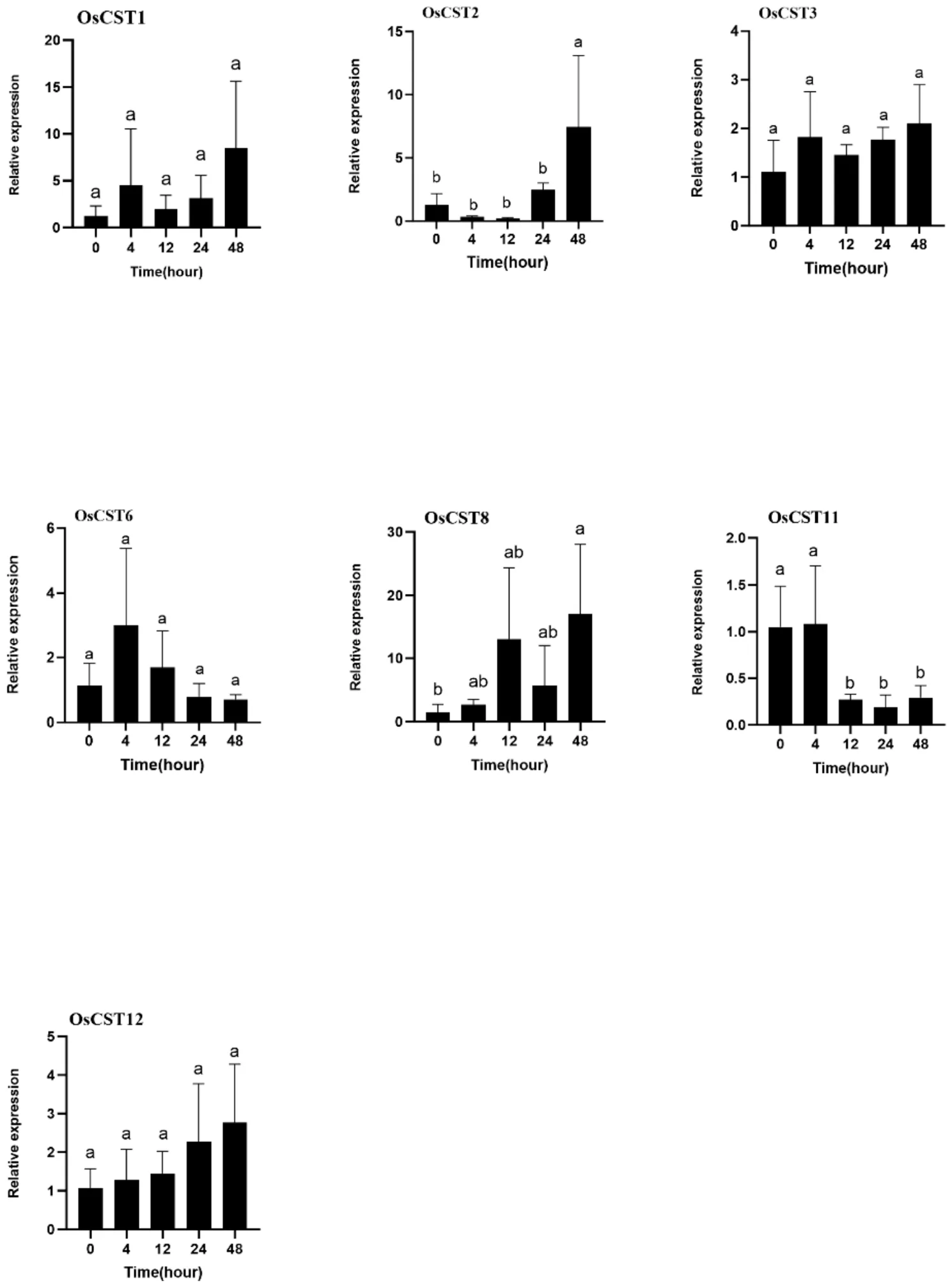
XMG rice seedlings with four fully expanded leaves were subjected to low-temperature treatment in a cooling chamber. Leaf tissues were harvested at 0, 4, 12, 24, and 48 h after low-temperature exposure. Transcript levels were determined by real-time quantitative PCR; relative expression was normalized to the 0 -h calibrator sample (0 h, set to 1.0). Each treatment contained three biological replicates. Error bars indicate the standard deviation (SD). One-way analysis of variance (ANOVA) was performed, followed by Duncan’s multiple-range test. Different lowercase letters indicate significant differences at *p* < 0.05.

Real-time quantitative PCR results revealed that cold stress induced transcript accumulation of *OsCST1*, *OsCST8*, and *OsCST12* in leaves to levels over five-fold higher relative to the 0 -h calibrator. Notably, *OsCST8* exhibited the strongest induction; its transcript abundance at 48 h of cold treatment reached 28-fold relative to the 0 -h calibrator. Furthermore, *OsCST11* was the only gene showing suppressed expression in leaves: its transcript levels began to drop after 12 h cold exposure, and at the 48 h time point, its mRNA abundance accounted for merely 10% of the 0-h calibrator. (Figure 3).

In roots, most genes exhibited expression changes under cold stress, whereas whereas significant down-regulation was observed for *OsCST8* under prolonged cold exposure. Transcript levels of *OsCST2*, *OsCST3*, *OsCST6*, and *OsCST12* were up-regulated. Among them, mRNA levels of *OsCST6* peaked at 24 h of cold treatment (10-fold higher relative to the 0 -h calibrator) and remained highly expressed thereafter. *OsCST2*, *OsCST3*, and *OsCST12* exhibited rapid cold-induced expression responses: their transcript levels were already elevated at 4 h post-treatment, after which expression gradually declined and returned to basal levels by 48 h.

**Figure 4:**
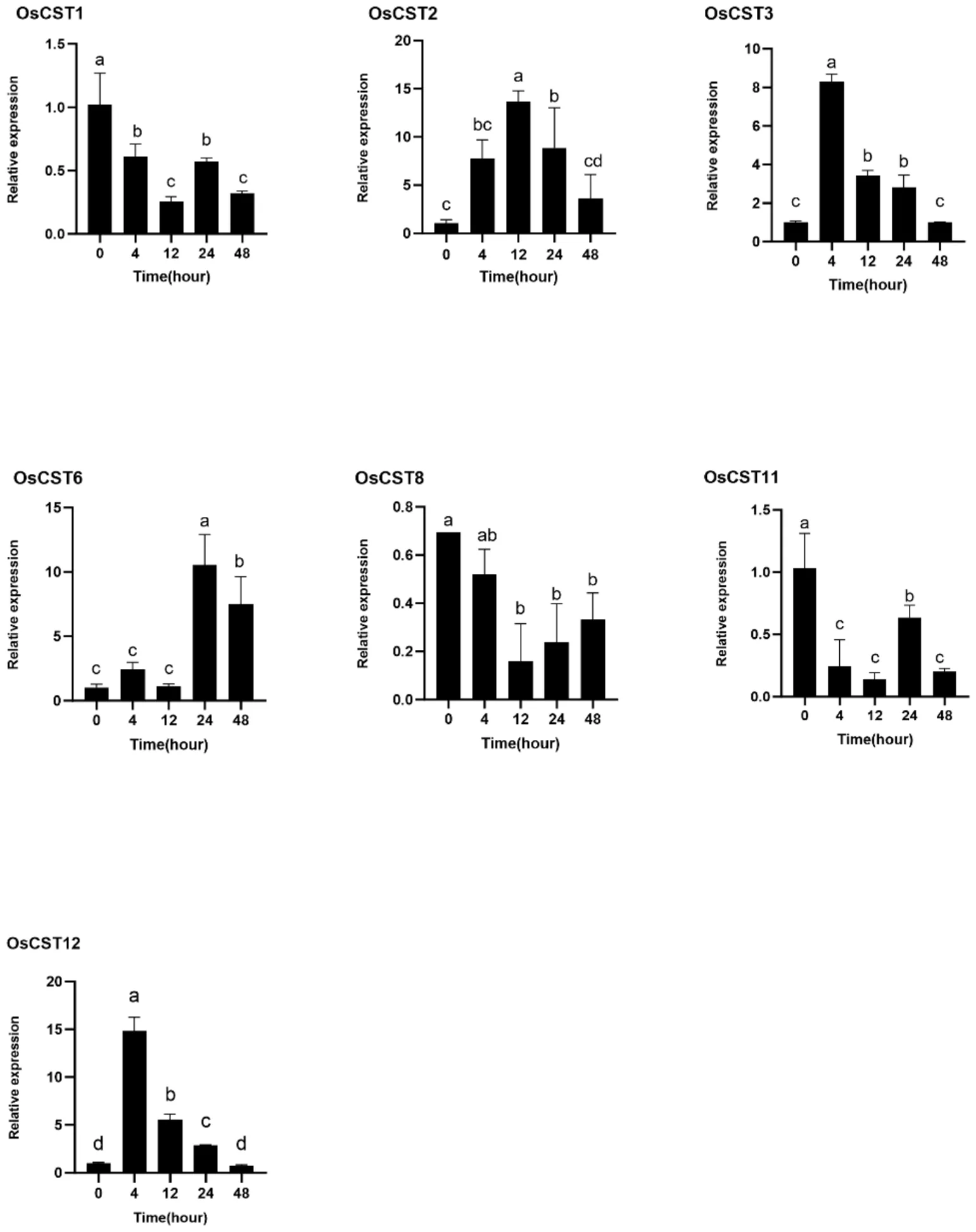
Expression patterns of phytocystatin family genes in roots under low-temperature stress. XMG rice seedlings with four fully expanded leaves were subjected to low-temperature treatment in a cooling chamber. Root tissues were harvested at 0, 4, 12, 24, and 48 h after low-temperature exposure. Transcript levels were determined by real-time quantitative PCR; relative expression was normalized to the 0 h calibrator sample (0 h, set to 1.0). Each treatment contained three biological replicates. Error bars indicate the standard deviation (SD). One-way analysis of variance (ANOVA) was performed, followed by Duncan’s multiple-range test. Different lowercase letters indicate significant differences at *p* < 0.05.

In contrast, *OsCST1* and *OsCST11* displayed reduced transcript abundance in roots under cold stress. Transcript levels of both genes dropped significantly after 4 h of cold exposure and stayed low throughout the treatment period. At the 48 h time point, the expression level of *OsCST1* was only 31.7% relative to the 0 h calibrator, while that of *OsCST11* reached merely 19.6% (Figure 4).

### 3.3. Transcriptional Response of Rice Phytocystatins Genes to ABA Treatment

**Figure 5:**
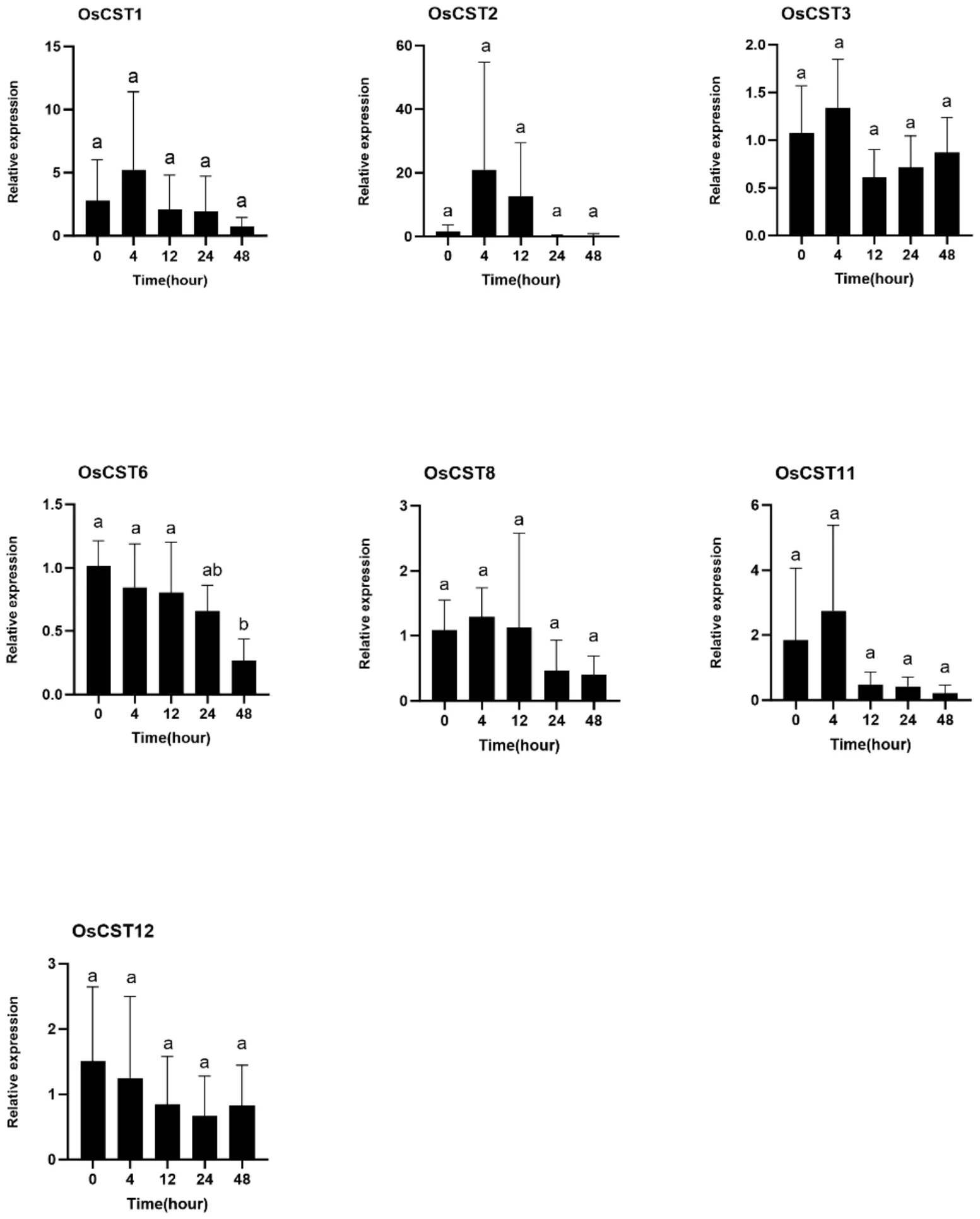
Expression patterns of phytocystatin family genes under ABA treatment. XMG rice seedlings with four fully expanded leaves were treated with 75 μmol L^−1^ ABA solution. Leaf tissues were harvested at 0, 4, 12, 24, and 48 h after treatment. Transcript levels were determined by real-time quantitative PCR; relative expression was normalized to the 0 h calibrator sample (0 h, set to 1.0). Each treatment contained three biological replicates. Error bars indicate the standard deviation (SD). One-way analysis of variance (ANOVA) was performed, followed by Duncan’s multiple-range test. Different lowercase letters indicate significant differences at *p* < 0.05.

Abscisic acid (ABA) is a plant hormone that acts as a critical signal molecule to regulate plant responses to cold stress [25]. To screen for ABA-responsive phytocystatin genes, we applied a five-fold change as a preliminary threshold for prominent transcriptional amplitude, together with statistical significance (*p* < 0.05). Three genes satisfied this combined screening criterion. These genes are regarded as ABA-responsive, although ABA responsiveness alone cannot prove their involvement in ABA-mediated cold-stress signaling. Transcript abundance of *OsCST2* increased at 4 h and 12 h after ABA treatment but declined at 24 h. The mRNA levels of *OsCST6* and *OsCST11* were reduced at 48 h after ABA treatment (Figure 5).

### 3.4. Transcriptional Pattern of Rice Phytocystatins Genes

Transcriptional profiles of rice phytocystatin genes across various tissues and developmental stages were analyzed using data retrieved from the Rice Expression Database (RED, https://ngdc.cncb.ac.cn/red/, accessed on 24 December 2025) [23]. Full metadata, including RED dataset identifiers, sampled tissues, developmental stages, locus-based gene IDs, and expression units, are provided in the Supplementary Materials. These public data originate from Nipponbare and not from the XMG (Xiaomagu) genotype used in this study.

**Figure 6:**
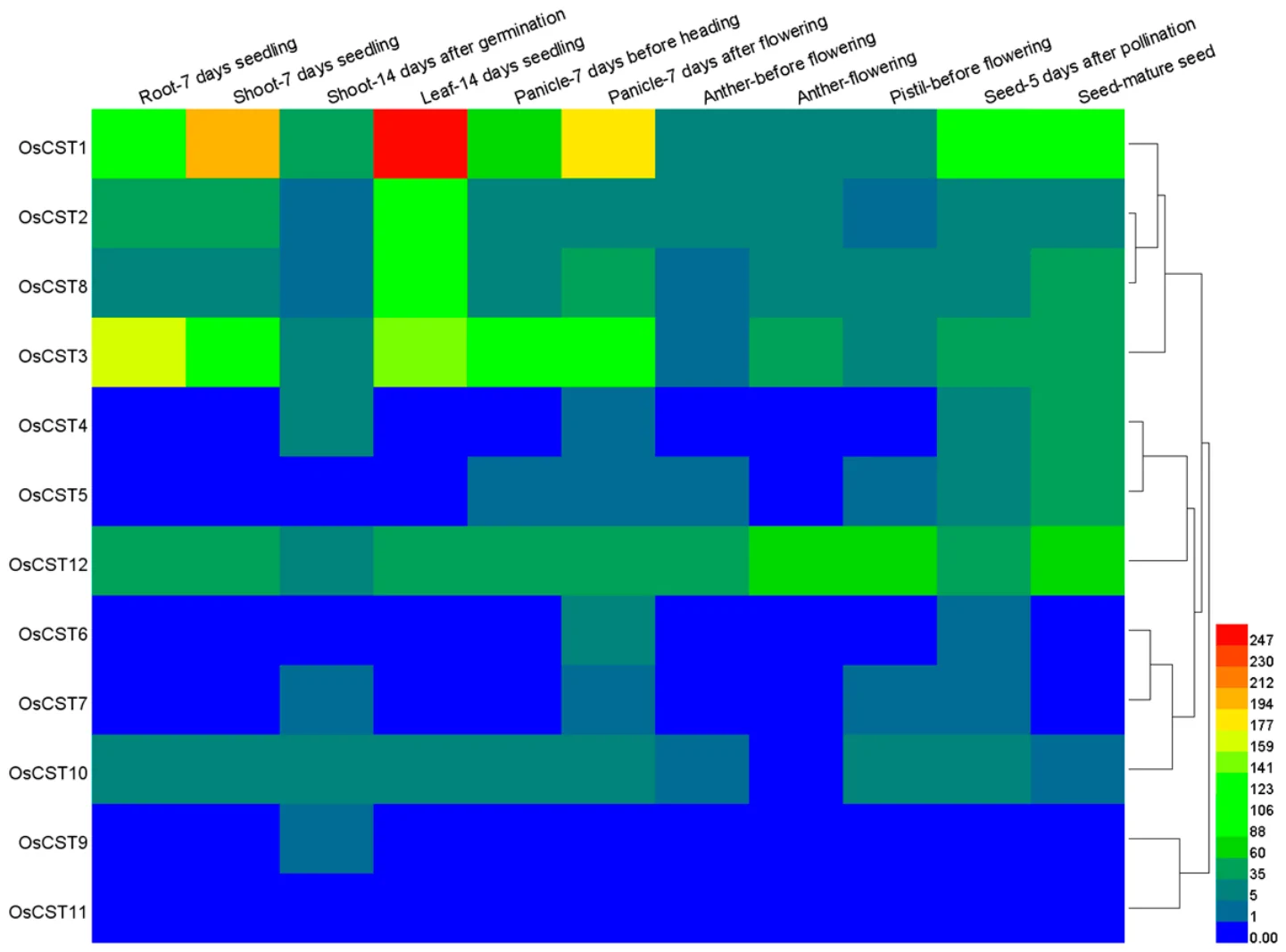
Heatmap of phytocystatin family gene expression across different rice tissues and developmental stages. Expression data for Nipponbare were downloaded from the Rice Expression Database (RED). These public-domain data are derived from Nipponbare. The heatmap was generated using Heml software1.0.

The results revealed that *OsCST1* and *OsCST3* maintained high transcript levels in all examined tissues and developmental stages; *OsCST2*, *OsCST8*, and *OsCST12* displayed moderate expression levels. Notably, *OsCST12* was the sole phytocystatin gene with abundant transcripts in reproductive organs including pistils and anthers. The remaining genes exhibited low overall expression and presented distinct tissue- or development-stage-specific expression patterns (Figure 6).

## 4. Discussion

Sequence comparison with the reference japonica rice cultivar Nipponbare revealed that *OsCST4* exhibits unique structural features distinct from other members of the rice phytocystatin family [26]. In contrast to the generally stress-responsive expression patterns of most *OsCST* genes, *OsCST4* transcripts were undetectable in both leaves and roots under cold treatment in the present study. Consistently, RED evidence also supports the extremely low transcript abundance of *OsCST4* in rice shoots, panicles, and seeds, indicating that this gene displays a constitutively low and tissue-restricted expression profile under normal and low-temperature conditions. The atypical sequence structure and extremely low transcription level suggest that *OsCST4* may possess functional characteristics different from typical stress-responsive phytocystatins. Considering its unique structural property and suppressed expression under cold stress, we speculate that *OsCST4* may undertake specialized biological roles that differ from other family members. Further systematic functional verification is required to clarify its precise physiological function and regulatory role in rice growth and stress adaptation.

It has been nearly 40 years since the first plant cystatin gene was isolated from rice seeds [27]; nevertheless, our knowledge of rice cystatin functions remains limited, particularly their in vivo regulatory roles. Numerous studies have demonstrated that reduced transcript levels of plant cystatins are closely correlated with elevated cysteine protease activities [28,29]. Transcriptional changes in phytocystatin genes under abiotic stress provide critical clues for understanding the modulation of plant proteolytic cascades [4]. Notably, the present study only characterizes transcript-level responses of rice phytocystatin family genes under cold and ABA treatments. Neither protein abundance nor cysteine protease enzymatic activity was determined in this work. Therefore, transcriptional alterations alone cannot serve as direct evidence for the functional regulation of proteolytic networks.

Previous studies have characterized the expression profiles of rice cystatin genes under multiple abiotic stresses, including high salinity and mechanical wounding. In comparison, the present study systematically profiled the transcriptional responses of the entire rice phytocystatin gene family to cold stress, providing a comprehensive foundation for further exploring the regulatory functions of these genes in plant proteolytic networks under low-temperature conditions.

Phytocystatin genes isolated from the cold-tolerant rice landrace Xiaomagu (XMG) exhibited distinct and tissue-specific transcriptional patterns under cold stress. Except for *OsCST11* and *OsCST12*, most family genes displayed tissue-dependent expression alterations. *OsCST12* was consistently up-regulated, while *OsCST11* was down-regulated in both leaves and roots under cold treatment. OsCST8 showed leaf-specific induced expression, whereas *OsCST2*, *OsCST3*, and *OsCST6* were significantly up-regulated only in root tissues. *OsCST1* exhibited opposite expression trends in different organs, with up-regulation in leaves and down-regulation in roots under cold stress. Combined with its constitutively high expression in various tissues, this organ-specific expression pattern suggests that *OsCST1* may execute distinct biological functions in different tissues during cold adaptation. Overall, these divergent transcriptional responses provide valuable candidate gene resources for subsequent functional verification.

Rice cold adaptation is modulated by multiple molecular pathways, including ABA-mediated signal transduction. ABA-responsive cis-regulatory elements have been identified in the promoter region of the Arabidopsis cystatin gene *AtCYSa*. Transgenic plants overexpressing the ABA-responsive element binding protein gene *AREB1* maintain high *AtCYSa* transcript levels under both normal and cold conditions [17]. In addition, ABA treatment also induces the expression of the wheat cystatin gene *TaMDC1* [15]. These findings demonstrate that plant cystatins can participate in cold stress responses through ABA-dependent signaling pathways. In this study, the transcript abundance of *OsCST6* and *OsCST11* decreased after ABA treatment, which is consistent with previous studies [19]. Among them, *OsCST11* was repressed by cold stress in both leaves and roots, making it a promising candidate for further functional research. However, the altered expression of phytocystatin genes under exogenous ABA treatment cannot directly confirm that their cold-responsive patterns rely on ABA signaling. Further verification using ABA-deficient or ABA-insensitive rice mutants is required to validate this regulatory mechanism.

*OsCST2* was the only family gene significantly induced by ABA treatment in the present study, which differs from the results of previous reports. This inconsistency may be attributed to differences in rice varieties or the developmental stages of experimental materials. Importantly, *OsCST2* (previously designated *OsCPI2*; Chromosome 5, NCBI accession: NM_001402581.1, NCBI gene locus: LOC4339190) has been confirmed to interact with the rice mature cysteine protease OsCysP7 in laboratory unpublished data [30]. The abundance of mature OsCysP7 protein rises under cold stress, and OsCysP7 knockout rice lines exhibit enhanced cold sensitivity (our unpublished data). Although these findings support the potential functional relevance of *OsCST2* in cold response, the current evidence is insufficient to draw definitive functional conclusions. Further transgenic and genetic validation experiments are essential to clarify the precise roles of these candidate genes in rice cold tolerance. The lack of comparative analysis with cold-sensitive rice genotypes and the absence of in vivo functional verification are the main limitations of this study, and further experimental work is required to elucidate the molecular mechanisms of these phytocystatin genes in rice low-temperature resistance.

## 5. Conclusions

Since the first plant cystatin gene was cloned, most studies on plant cystatins have primarily focused on their defensive roles against herbivores and pathogens [31]. In contrast, investigations addressing their endogenous physiological functions remain limited. In the present study, we demonstrated that rice phytocystatin family genes exhibit divergent transcriptional responses under cold stress. Multiple key questions remain to be clarified, especially regarding the endogenous proteolytic regulatory functions of rice phytocystatins during rice cold adaptation.

## Data Availability

The original contributions presented in this study are included in the article/Supplementary Material. Further inquiries can be directed to the corresponding author.

## Supplementary Materials

The following supporting information can be downloaded at: https://www.mdpi.com/article/10.3390/genes17091037/s1, Supplementary File S1: Raw data downloaded from the RED database. Table S1: Information of specific primer pairs for 12 rice phytocystatin family genes designed based on NCBI-retrieved sequences. Table S2: List of all qPCR primer sequences used in this study. Table S3: Tentative gene symbols and deduced protein sequences of phytocystatin genes from rice landrace XMG (Xiaomagu).

## Abbreviations

The following abbreviations are used in this manuscript:

ABA	Abscisic acid
AREB1	ABA-responsive element binding protein
bp	Base pair
cDNA	Complementary DNA
Ct	Cycle threshold
DNA	Deoxyribonucleic acid
FDH	Formate dehydrogenase
gDNase	Genomic DNA deoxyribonuclease
mRNA	Messenger RNA
NCBI	National Center for Biotechnology Information
PCR	Polymerase chain reaction
qPCR	Quantitative polymerase chain reaction
RED	Rice Expression Database
ROX	Reference dye
RT	Reverse transcription
RT-PCR	Reverse transcription polymerase chain reaction
XMG	Xiaomagu
